# Convalescence at the Sea-Side

**Published:** 1902-03-29

**Authors:** 


### The Hospital

A Journal of

The Medical Sciences and Hospital Administration.

Vol. XXXI.—No. 809.] Edited by Sir Henry Burdett, K.C.B., and Solomon C. Smith, M.D., M.R.C.P. [March 29, 1902.

### CONVALESCENCE AT THE SEA-SIDE.

At this time of year the English South Coast becomes a veritable convalescent institution whither the human wreckage of the winter foregathers in the hope of re-establishing its scattered forces and regaining health and energy with which to face the work and worry of the world once more. There may, perhaps, be some doubt as to the preponderating advantages of the sea-side as a permanent residence for invalids. It is not every sick man who can sleep when within the influence of the tides; neurotics often suffer much, their natural irritability of constitution being liable to many perturbations at the hands of the ever-changing weather; while, in regard to anæmia and its various manifestations, although a short visit to the sea-side works wonders, a longer stay, especially when the residence is situated near the sea level, is apt to produce the very malady we wish to cure. Indeed, in the choice of a residence, notwithstanding the manifest tendency shown by chronic invalids to crowd into sea side health resorts, there are many factors which are of far greater importance than sea air.

As a means, however, of hastening canvalescence after acute disease, of affording free play to those vital forces on the orderly operation of which health depends, and of giving that "fillip" which is often needed, even by those with the strongest constitutions when they have been pulled down by illness, to lift them out of the "rut" of invalidism and set them on the road to health, there is nothing like sea air, and in the late winter and early spring there is no place in England like the South Coast.

Ours is not the pen to describe the charms of the sudden change from fog to sunshine or the contrast between the stuffiness of town and the glorious expanse of sea and air which a couple of hours' run enables the convalescent Londoner to obtain, but we may point out how effectual it is as a therapeutic agent. The condition of torpidity into which even the healthiest frame may sink after severe illness is due in a large degree to the absence or the derangement of the accustomed stimuli by which our inner mechanism is wont to be set in motion. One may almost say that the better the nursing the greater the difficulty of finding the stimulus which is necessary for the initiation of convalesence. With everything supplied, and every want anticipated, the invalid remains in a rut of undesire. Here it is that the change to the sea-side once more sets the wheels of life in action. Nothing can demonstrate more conclusively the reflex influence of change and the good effect of stirring up the dull monotony of illness by the introduction of new stimuli, than the wonderful restoration of appetite which in some cases occurs almost immediately on arrival at the sea-side. We are referring here not to cases of organic disease but to convalescence in persons of sound constitution who have been knocked down by illness and have failed to find their feet again under ordinary home treatment. Under the new conditions appetite returns, exercise is taken, hours a day are spent in the open air, and in a curiously short space of time life is looked at from quite a new point of view. What then are the drawbacks to sea-side convalescence during the early spring. They are not many, but they deserve careful consideration:—Dyspepsia, sleeplessness, exhaustion from doing too much, and (especially after influenza) pneumonia and various internal congestions from undue exposure. The latter are generally the penalty of leaving home too soon; for the sea-side, even our South Coast, is full of dangers to the over-bold. Many times in the course of a day may one pass from summer to winter, and, wearying as it may seem, it is essential that the convalescent should always be clad in woollen and should always carry wraps. The "biliousness" of the seaside is no doubt to a large extent the result of over-eating. Appetite overruns digestive power, and unless an occasional "dose" be taken dyspepsia is soon produced. Again, it often is the result of alcohol. When days are fine the question of alcohol does not so much intrude itself, but during spells of wet weather smoke rooms and billiard rooms are much frequented, whisky and soda is the order of the day—and then we are told that sea air leads to biliousness! Let the convalescent hold a steady rein upon his appetite. Sleeplessness is a more difficult matter. To some the sough of the sea is a lullaby which brings sleep at once, and on the strength of the eulogies of these happy people the hotel and lodging-house keepers charge high prices for "front rooms." But to many neurotic persons the angry beating of the waves and the blasts of the south-westerly gales, which sometimes rise with curious suddenness, are full of terrors and are quite enough to drive away all tendency to sleep. Convalescents should never forget that sound natural sleep is in itself a most important means to health. A bedroom, then, should be chosen not for the beauty of its aspect, but for its fitness as a sleeping place, for unless one can obtain a due allowance of "nature's sweet restorer" all other aids to convalescence will be thrown away. After all, however, the chief risks in convalescence at the sea-side are eating too much, doing too much, and especially daring too much in the way of exposure to cold.

